# Comparison of Therapy Practice at Home and in the Clinic: A Retrospective Analysis of the Constant Therapy Platform Data Set

**DOI:** 10.3389/fneur.2019.00140

**Published:** 2019-02-25

**Authors:** Jason Godlove, Veera Anantha, Mahendra Advani, Carrie Des Roches, Swathi Kiran

**Affiliations:** ^1^The Learning Corporation, Newton, MA, United States; ^2^Aphasia Research Laboratory, Speech Language and Hearing Sciences, Boston University, Boston, MA, United States

**Keywords:** aphasia, stroke, technology, rehabilitation, clinic

## Abstract

**Background:** Computer-based therapies can provide an affordable and practical alternative by providing frequent intervention for stroke survivors with chronic aphasia by allowing the opportunity for home exercise practice, however more evidence is needed. The goal of this retrospective analysis was to compare the time course of therapy engagement when therapy was targeted in the clinic or at home by post-stroke individuals. We examined if home users of the therapy were compliant in therapy and if this documented practice time was associated with improved outcomes similar to clinic patients who practiced under the guidance of a clinician.

**Methods:** A retrospective analysis of anonymously aggregated data collected for 3,686 patients with post-stroke aphasia over the course of four years (2013–2017) was conducted. Participants either received therapy delivered through Constant Therapy only at home (*N* = 2,100) or only in the clinic (*N* = 1,577). Constant Therapy includes over 70 evidence-based therapies for language and cognitive skills. This program was individualized for each patient with targeted tasks that dynamically adapted to each individual's progress.

**Results:** Patients with <60% accuracy were analyzed to determine how long it took them to reach >90% accuracy. Results showed that both home-therapy and clinic patients reached 90% accuracy on their tasks similarly (Median = 3 sessions), but the frequency of therapy was significantly different with 50% of home users receiving therapy at least every 2 days while 50% of clinic patients only had therapy once every 5 days (*p* < 0.001). Thus, home-therapy users were able to master tasks in a shorter time (median of 6 days) than clinic patients (median of 12 days) (*p* < 0.001).

**Conclusion:** Outcomes of treatment are similar for home users and clinic patients indicating the potential usability of a home-based treatment program for rehabilitation for post-stroke aphasia.

## Introduction

It is estimated that ~100,000 persons acquire aphasia each year in industrial countries (eso-stroke.org). In addition, there are roughly 1.7 million new instances of brain injury each year. Even though aphasia is more debilitating than other disabilities (1, 2), rehabilitation for chronic aphasia is quite fragmented. After a limited number of speech therapy sessions immediately following stroke, persons with aphasia (PWA) receive little to no support to maintain progress or carry therapy strategies over into daily communicative activities. This negatively influences their quality of life as compared to individuals with other chronic disabilities (3, 4).

Research indicates that aphasia rehabilitation improves language and communication ability (5–9) but there are no clear standard of care guidelines for providing effective and efficient rehabilitation services for chronic aphasia. Approaches to improve specific aspects of language are effective but these treatments require one-on-one (clinician-patient) intervention with periodic visits to the hospital and clinic. Additionally, the frequency of clinic visits is determined by health insurance plans, the individual's mobility and access to transportation, as well as vicarious factors, including the geographic location of the hospital and adverse weather. These barriers complicate the already difficult path for consistent and systematic aphasia rehabilitation for chronic stroke survivors. A recent study in the United Kingdom documented that chronic stroke survivors received between 3 and 5 60 min therapy sessions over a period of 3 months (10), which is far lower than the recommended clinical practice recommendations (11).

One potential solution is to facilitate patient practice of rehabilitation at home. Several studies have examined computer based interventions at home and in the clinic, but most of these have included small sample sizes and the degree of outcomes have been variable (12–31). Some of these studies have incorporated different types of homework into their therapy program, and the amount of practice at home has varied greatly, partly contributing to different outcomes. Additionally, very few of these studies directly compared intervention at home vs. in the clinic, thus making it difficult to draw conclusions about whether therapy at home is equivalent to therapy provided in the clinic [see (32) for a full discussion on this topic].

One of the studies noted above provided preliminary efficacy for a remotely delivered cloud-based rehabilitation program called Constant Therapy (27) in 51 patients with aphasia. Experimental and control patients in this study practiced therapy tasks on an iPad, but experimental patients also practiced independently at home, while control patients only received therapy in the clinic. Results showed that experimental patients showed significantly more improvements on standardized tests than control patients, but they also received much more practice than control patients. Importantly, more severe patients showed more improvements than less severe patients. However, the experimental patients still received treatment in the clinic, so this study does not provide a direct comparison of clinic-only and home-only therapy.

To summarize, computer-based treatments are emerging as an alternative to delivering therapy for patients, however, for these types of treatment deliveries to become realistic alternatives to one-on-one in-clinic therapy visits, more evidence is required. The goal of this retrospective analysis was to compare the time course of therapy engagement and corresponding outcomes when therapy was targeted in the clinic or at home by post-stroke individuals. In both scenarios, patients practiced a computer-based therapy, however, therapy performed under the real-time guidance of a clinician was compared with therapy performed at home without real-time clinician guidance. We examined how often home users utilized the therapy and if this documented practice time was associated with improved outcomes similar to clinic patients who practiced under the guidance of a clinician. The hypothesis was that both home users and clinic users will improve in a similar timeframe on the tasks assigned in treatment and demonstrate the feasibility of a home-based treatment program for rehabilitation for post-stroke aphasia.

## Methods

### Participants

Over a span of 4 years (2013–2017), data was anonymously aggregated and analyzed from 20,000 independent PWA, all of whom presented with language and/or cognitive disorders. Constant Therapy was the software used for therapy delivery and is available for download on the iTunes and Google stores. Either clinicians could set up an account for the patient, or the patient could create an account after downloading the program from their application. Before the patients could sign into the account, they were presented with a written description of the user license agreement where they had to consent to the use of their exercise and therapy performance for scientific and research purposes. They were asked to provide information about their demographics, including age (in years) and years since injury (0–6 months, 6 months−1 year, 1–2 years, 2–5 years, 5–10 years, and more than 10 years). They were also asked to self-select which domains of the therapy they felt they needed improvement. Only two groups are analyzed for this paper, PWA who received therapy only when they were in the clinic (*n* = 1,577), and PWA who received therapy only while at home (*n* = 2,100). PWA who received therapy both at home and in the clinic were excluded from this study. Refer to Table 1 for a breakdown of age and years since injury for home and clinic PWA.

**Table 1:** Count of PWA in age and years since injury bins, by home and clinic therapy use.

**Age**	**10–20**	**21–50**	**51–70**	**71–100**	
Home	23	480	1,090	507	
Clinic	17	241	764	573	
Total	40	721	1,836	1,080	
**Years since injury**	**0–0.5**	**0.5–1**	**1–2**	**2–5**	**>10**
Home	1,052	340	275	254	61
Clinic	936	274	165	139	23
Total	1,988	614	440	393	84

This project was considered an IRB exempted retrospective analysis by Pearl IRB (#17-LNCO-101) under 45 CFR 46.101(b) category 2.

### Therapy Program

Data was collected using a mobile therapy platform, Constant Therapy, which includes 70 evidence-based tasks with varying levels of difficulty ranging from one level to 10 levels, for a total of 244 individual task levels. The tasks fall in the domains of language: (a) naming, (b) comprehension, (c) speaking, (d) reading, and (e) writing; and cognitive skills: (a) attention, (b) executive skills and problem solving, (c) mental flexibility, (d) memory, and (e) visuospatial skills. Patients practiced in the clinic with clinicians (i.e., clinic group) or independently if they were not currently receiving therapy (i.e., home group). This program was standardized, in that all tasks were designed and administered in a uniform way, but also individualized in that patients were assigned tasks that addressed their individual strengths and weaknesses and dynamically adapted to each individual's progress.

The data presented here are curated from a larger data set. To keep the analyses between the home and clinic users comparable, several filters were applied to the data. Thus, for the purposes of this study, only the tasks where PWA were initially struggling (<60% accuracy) and proceeded to then master the task (90% accuracy and greater) were included in the analyses. This method of selecting data also equated the clinic and home patient groups on factors such as overall severity. Second, only instances where PWA took more than 1 day but fewer than 60 days to master the task were included. In addition, only tasks that at least 15 clinic-only PWA and 15 home-only PWA completed were included for analysis, which left 46 task levels with enough data to be analyzed (see Table 2 for a count of subjects included in each task level) across 34 unique tasks. We should note that selection of these 46 tasks does not imply that for the tasks not selected for analysis, there are preexisting differences in the performance between the two groups. In fact, clinicians did not chose the task at that difficulty level in the clinic for 116 task levels and 80 tasks levels were not assigned to both groups (presumably because these tasks were at the highest or lowest difficulty levels). Only 2 of the task levels had enough in clinic use but not enough at home use, the Symbol Matching level 1 and Picture Matching level 1 tasks, both of which are only assigned at home to the most severe cases after home users have failed at the level 2 version of those tasks, respectively, which rarely happens. Thus, if the tasks were used in the clinic, they were included in the study.

**Table 2:** Count of PWA included in each task by home and clinic therapy use.

**Task name and level**	**Count of clinic PWA**	**Count of home PWA**	**Total**
Auditory command 1	49	94	143
Auditory command 1^*^	40	44	84
Auditory command 2	67	220	287
Auditory command 2^*^	42	120	162
Auditory command 3	17	74	91
Calendar 1	41	99	140
Category identification 1	54	212	266
Category matching 1	86	198	284
Clock math 1	92	88	180
Clock math 2	91	127	218
Clock math 3	30	104	134
Clock reading 1	92	28	120
Clock reading 2	22	45	67
Currency 1	20	27	47
Feature matching 1	36	197	233
Flanker 1	36	62	98
Functional math 1	24	39	63
Functional reading 1	59	84	143
Functional reading 2	22	66	88
Instruction sequencing 1	111	116	227
Letter to sound matching 1	26	119	145
Map reading 1	38	239	277
Mental rotation 1	21	56	77
Minimal pairs - same or different 1	20	23	43
Pattern recreation 1	49	27	76
Pattern recreation 2	29	48	77
Pattern recreation 3	22	36	58
Picture matching 2	52	88	140
Picture naming 1	59	46	105
Picture naming 1^*^	74	71	145
Picture naming 3	20	54	74
Picture N-back memory 1	135	390	525
Picture N-back memory 2	41	130	171
Playing card slapjack 1	134	295	429
Reading passage 1	28	65	93
Short reading 1	38	56	94
Sound identification 1	34	354	388
Sound to letter matching 1	17	101	118
Spoken word comprehension 1	33	41	74
Symbol matching 6	28	121	149
Voice mail 1	125	143	268
Voice mail 2	65	124	189
Word identification 1	54	98	152
Word problem 1	24	63	87
Word repetition 1	32	45	77
Written word comprehension 1	17	27	44

* *Denotes newer versions of a task that were updated and therefore treated as a separate task*.

### Data Analysis

Five dependent variables were considered:

- The *number o*f calendar *days* passed from when users first struggled with a task (with an accuracy of 60% or less) to when they first succeeded at the task (with an accuracy of 90% or more). This measures how many days passed as the users practiced a particular therapy task before improving,
- The *median* number of *days between each ther*apy session during that period to gauge how frequently therapy was performed while excluding events like vacations and missed therapy days,
- *The* number of *days* of therapy the user required in order to succeed at a task,
- The *number of items p*er therapy *day* that users completed, and
- The *total number of th*erapy items (individual questions or exercises) for users to reach 90% accuracy.

To provide a context for these variables, if a user failed at a math task on Monday, practiced again on Wednesday, and succeeded at the task on Friday, it took 3 days of therapy spread out over 5 calendar days with 2 days between each therapy session in order to succeed at the task. If the patient practiced 5 items per therapy day, then the total number of items required to succeed at the task would be 15.

**Figure 1:**
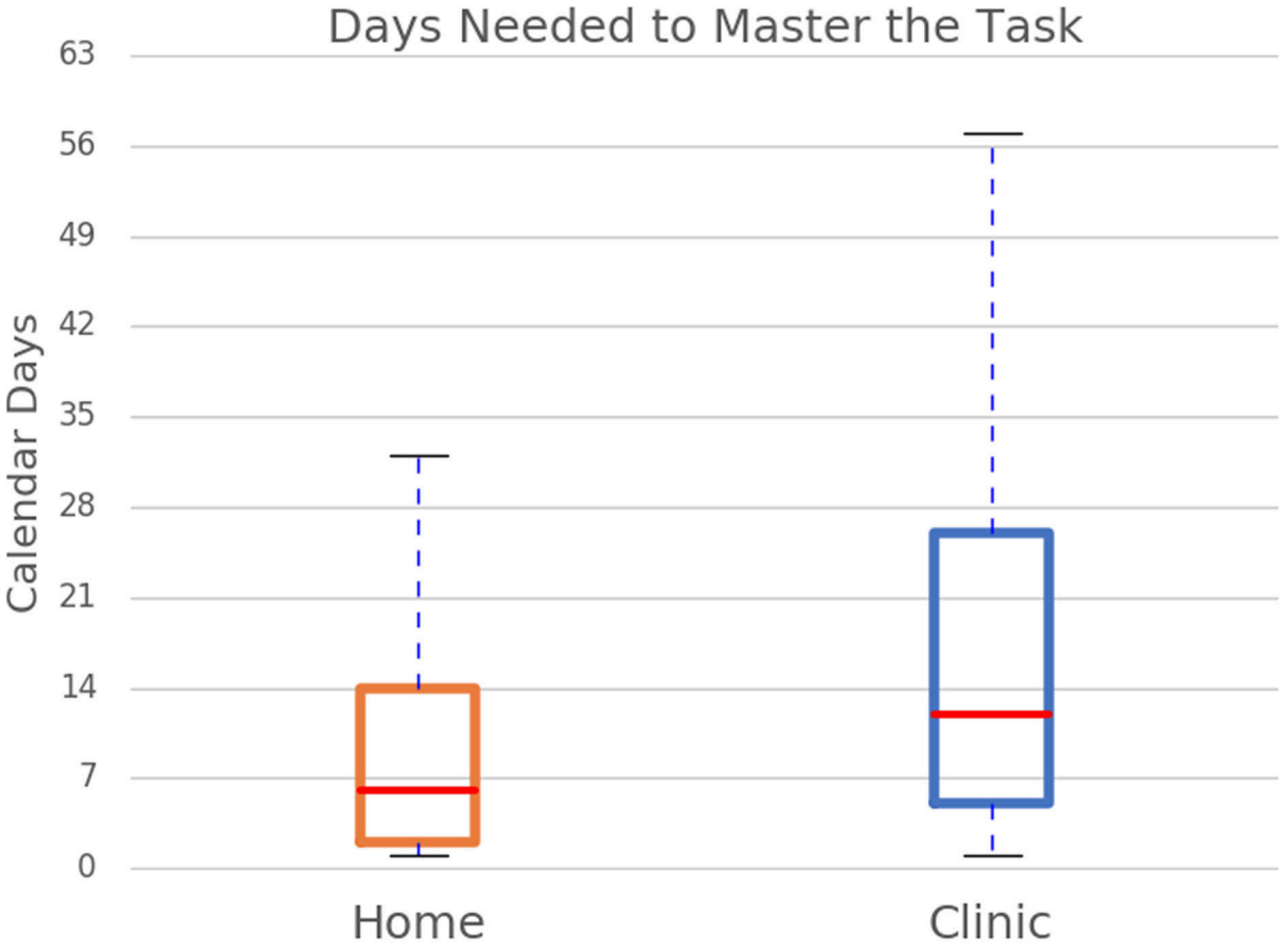
Summary of median calendar days needed to master the task by user group.

With the exception of the median number of days between therapy sessions and the number of items per therapy day, all of the measures had exponential distributions (Figure 1). Therefore, log transformations were performed and used for analyses with number of calendar days, number of days of therapy sessions, and total number of items. Statistics were completed using Statistical Package for the Social Science (SPSS Inc., Chicago, IL).

## Results

Data for 3677 PWA were analyzed for this study. The average age of home users was 59.77 and clinic users was 64.48 years. A *t*-test (since the age was entered as a continuous variable) (*t* = 9.03, *p* < 0.001) showed these differences were significant even though they were very similar. The average time since injury for home users was 2.01 years while clinic users was 1.59 years. A Mann–Whitney test was conducted because time since stroke was provided as a semi-categorical variable and this was also significantly different, Mann–Whitney (statistic = 1,455,696.0, *p* < 0.001). Given that there were pre-existing differences between the two groups in their age and time since stroke, these factors were entered as covariates into the data analyses.

**Figure 2:**
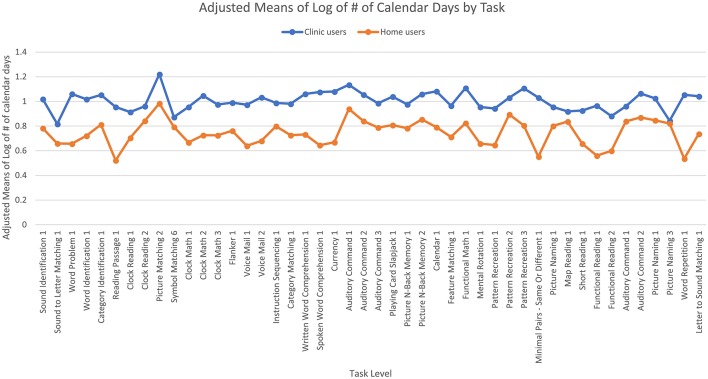
Adjusted means for the number of calendar days passed by user group. Covariates appearing in the model are evaluated at the following values: years since injury = 2.10, age = 61.62.

A two-tailed Pearson correlation revealed that all five measures were significantly correlated with each other (Table 3). Therefore, separate univariate ANCOVAs (instead of one MANCOVA) were run with each of the measures as the dependent variable and with the user group (clinic or home users) and the task (different tasks and different levels of task) as independent variables, controlling for age and time since injury. The first ANCOVA examined the log of the number of calendar days for users to reach 90% accuracy and found no significant effect of age, but there was a significant effect of years since injury [*F*_(1,7056)_ = 3.85, *p* < 0.05] where home users had a higher number of years since injury than clinic users. As noted above, there is a slight difference in time since injury between the two groups. However, a regression showed that time since injury did not significantly affect the log number of calendar days (*t* = −0.77, beta = −0.01, *p* = 0.44). There was a main effect of user group [*F*_(1,7056)_ = 279.44, *p* < 0.001] where clinic users took a higher number of calendar days to reach 90% accuracy than home users (Figure 2). Specifically, the adjusted mean log number of calendar days for users to reach 90% accuracy was 1.00 (SE = 0.01) for clinic users and 0.75 (SE = 0.01) for home users; in other words, the median number of calendar days was 12 for clinic users and six for home users. There was also a main effect of task level [*F*_(45,7,056)_ = 2.52, *p* < 0.001] where the number of calendar days for users to reach 90% accuracy varied across task levels. The interaction was not significant.

**Table 3:** Correlation results for log of number of calendar days, log of total number of items, log of number of therapy days, and median days between sessions.

		**Log of number of calendar days**	**Median days between sessions**	**Log of number of therapy days**	**Items per day**	**Log of total number of items**
Log of number of calendar days	r		0.51^***^	0.49^***^	0.05^***^	0.38^***^
	N		7,150	7,150	7,150	7,150
Median days between sessions	r			−0.24^***^	−0.12^***^	−0.22^***^
	N			7,150	7,150	7,150
Log of number of therapy days	r				0.11^***^	0.76^***^
	N				7,150	7,150
Items per day	r					0.68^***^
	N					7,150

*r, Pearson correlation; N, number of subjects across all 46 task levels*;

*** *p < 0.001*.

**Figure 3:**
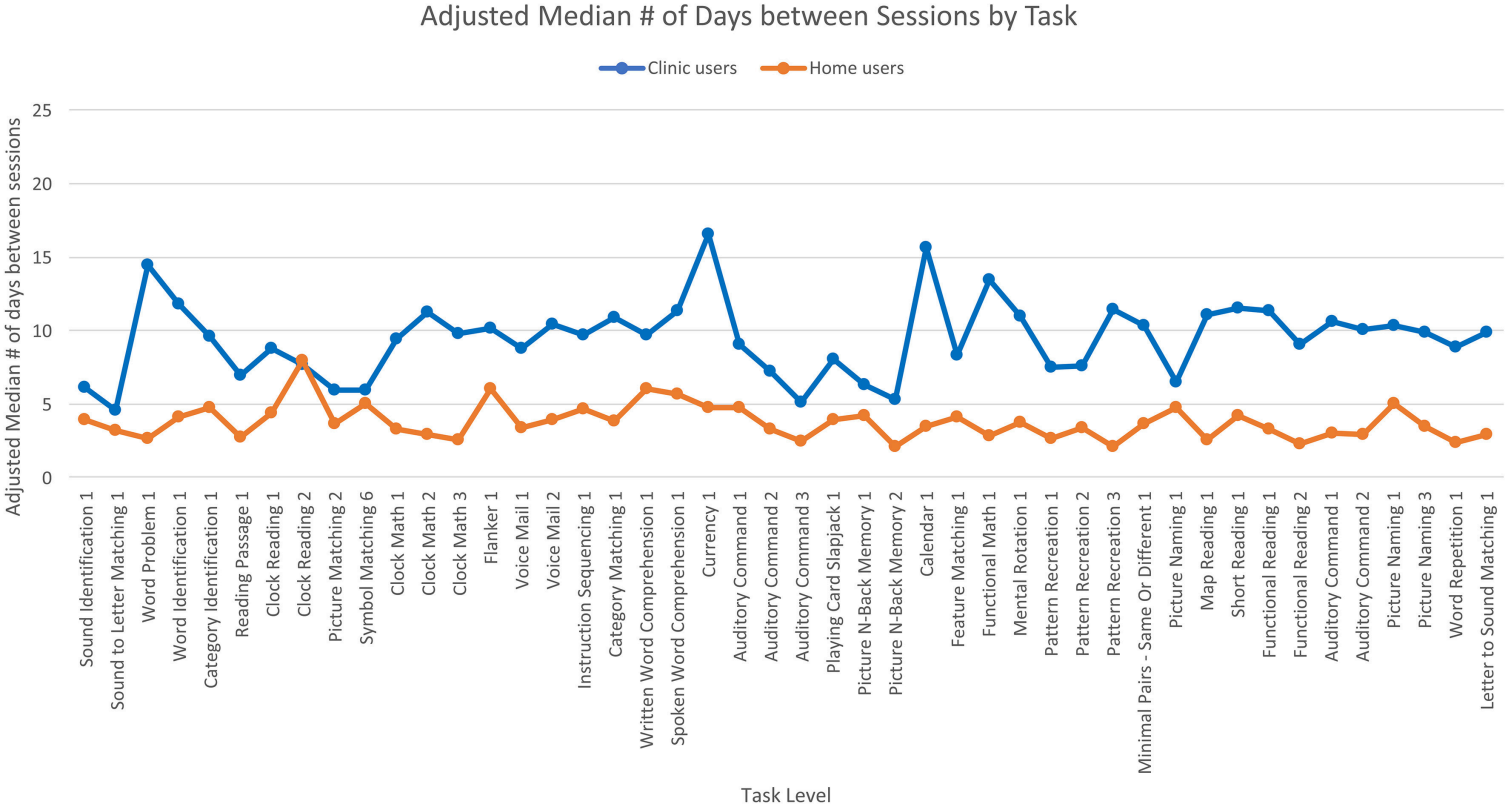
Adjusted means for the median number of days between each therapy session by user group. Covariates appearing in the model are evaluated at the following values: years since injury = 2.10, age = 61.62.

The second ANCOVA looked at the median number of days between therapy sessions and showed no significant effect of age or years since injury. There was a significant main effect of user group [*F*_(1,7,056)_ = 205.84, *p* < 0.001] where clinic users had a greater median numbers of days between sessions than home users (Figure 3), such that the median number of days between therapy sessions was five for clinic users and two for home users. The main effect of task level was not significant, but the interaction was significant [*F*_(45,7,056)_ = 2.97, *p* < 0.001] where clinic users had a greater median number of days between sessions than home users for some task levels but the opposite was true for other task levels.

**Figure 4:**
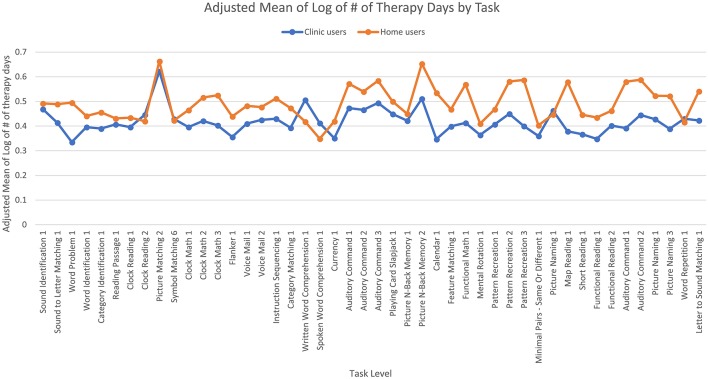
Adjusted means for the number of days of therapy the user completed by user group. Covariates appearing in the model are evaluated at the following values: years since injury = 2.10, age = 61.62.

The third ANCOVA examined the log of the number of therapy days that users took to reach 90% accuracy and found a significant effect of age [*F*_(1,7,056)_ = 9.18, *p* < 0.01] where home users were younger than clinic users. However, a regression showed that age did not predict the log number of therapy days (*t* = 0.57, beta = 0.01, *p* = 0.57). There was no significant effect of time since injury. There was a significant main effect of user group [*F*_(1,7,056)_ = 75.64, *p* < 0.001] where overall, clinic users took fewer days of therapy to reach 90% accuracy than home users (Figure 4). Specifically, the adjusted mean log number of days of therapy for users to reach 90% accuracy was 0.42 (SE = 0.01) for clinic users and 0.49 (SE = 0.004) for home users; the median number of days of therapy was two for clinic users and three for home users. There was also a significant main effect of task level [*F*_(45,7,056)_ = 3.30, *p* < 0.001] where the number of days of therapy differed across task levels. The interaction was also significant [*F*_(45,7,056)_ = 1.99, *p* < 0.001] where clinic users required more days of therapy than home users for some task levels but the opposite was true for other task levels.

**Figure 5:**
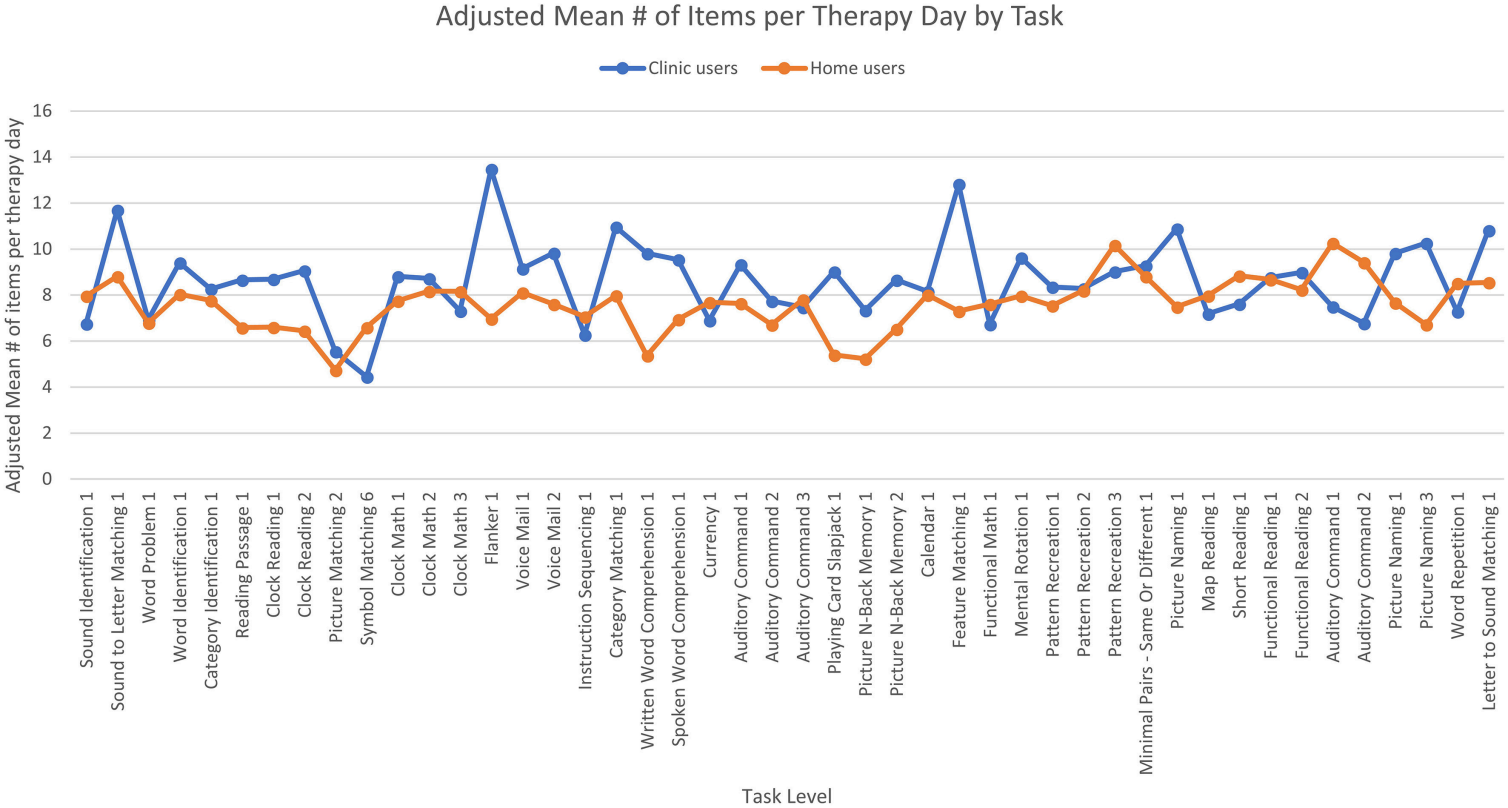
Adjusted means for the number of items per day the users completed by user group. Covariates appearing in the model are evaluated at the following values: years since injury = 2.10, age = 61.62.

The fourth ANCOVA examined the number of items per day that users completed and found no significant effect of age or time since injury. There was a significant main effect of user group [*F*_(1,7,056)_ = 13.44, *p* < 0.001] where clinic users completed more items per therapy day than home users. Specifically, the adjusted mean number of items per therapy day that users completed was 8.66 (SE = 0.11) for clinic users and 7.60 (SE = 0.08) for home users; the median number of items per therapy day was 8.17 for clinic users and 6.40 for home users. The main effect of task level was not significant, but the interaction was significant [*F*_(45,7,056)_ = 5.93, *p* < 0.001] where clinic users completed more items per therapy day than home users on some task levels while the opposite was true for other task levels (Figure 5).

**Figure 6:**
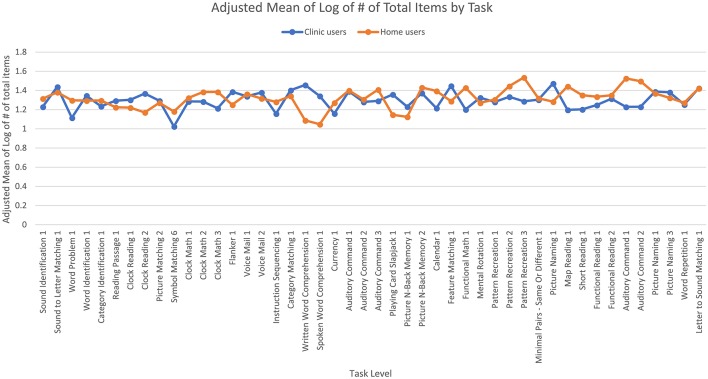
Adjusted means for the total number of therapy items the users completed by user group. Covariates appearing in the model are evaluated at the following values: years since injury = 2.10, age = 61.62.

The fifth ANCOVA looked at the log of the total number of items that users took to reach 90% accuracy and found no significant main effects, nor any effects of age or time since injury. In this case, the adjusted mean log total number of items for users to reach 90% accuracy was 1.30 (SE = 0.01) for clinic users and 1.32 (SE = 0.01) for home users; the median total number of items was 20 for both clinic and home users. The interaction between user group and task levels was significant [*F*_(45,7,056)_ = 5.38, *p* < 0.001] where clinic users required more items than home users for some task levels and the opposite was true for other task levels (Figure 6).

## Discussion

The goal of this retrospective analysis was to compare the time course of therapy engagement when therapy was practiced in the clinic or at home by post-stroke individuals. The main observation of the study was that to improve on a specific task, it is the amount of practice that is required rather than the setting that is practiced. Also, patients take advantage of practicing multiple times a week if they have the option, and are able to engage with the software independently.

First, there was no significant difference between clinic and home users on the total number of items that were required to reach 90% accuracy (ANCOVA #5), although there was a significant interaction between user group and task level (with some tasks requiring greater items practiced compared to others). Also, clinic users needed significantly fewer days of therapy to reach 90% accuracy than home users (ANCOVA #3). Again, there was a significant effect of task level and the interaction was also significant, though the effect where clinic users required fewer days than home users was the most prevalent effect, with six of the 46 task levels showing the opposite effect. One element of this effect can be explained through the analysis which showed that clinic users practiced more items per therapy day than home users (ANCOVA #4) although again the interaction effect indicated some tasks required greater items practiced compared to others. These three results indicate that while the clinic users practice more items per therapy day, there are no differences in the overall number of items practiced across the two groups. These results appear to suggest that post-stroke patients (both home and clinic users) require the same number of items to achieve criterion on a given task. This observation is important because it demonstrates that patients who use the software, are getting benefit from the therapy independently, and this is not significantly different from the benefit gained from users who are working with the guidance of a clinician.

Importantly, there are differences between the clinic and home users (ANCOVA #1); clinic users take significantly more calendar days to reach 90% accuracy than home users. Though there was also a significant effect of task level, the interaction was not significant, so the two groups did not differ significantly based on the task level. Additionally, the results showed that the clinic users had a significantly greater number of days between therapy sessions than home users (ANCOVA #2). The interaction was significant this time, though the main effect of clinic users having more days between sessions was true for all but four of the 46 task levels. Both these findings suggest that clinic users take longer than home users to reach 90% accuracy due to accessibility to the program. Users who only used the therapy program in the clinic would need to wait at least until their next therapy session to do the task again. On the other hand, home users had access to the therapy program every day at home. Thus, they had the option to practice therapy multiple times a week.

The main effect of user group showed that while home users complete their therapy quicker than clinic users, the clinic users do not need as many days of therapy to reach 90%. Although this observation seems contradictory, it is possible that clinic users may benefit from one-on-one clinician interaction to master the therapy tasks relative to home users who may or may not have family support to complete the therapy tasks.

It is meaningful to put these results in context and comparing median values presented in the results illustrate the differences nicely. Say that a clinic-only user were to start therapy on the first of the month; the results show that it takes this user 12 calendar days to progress from 60 to 90% performance on a task. This person attends therapy sessions every 5–7 days but needs two sessions to reach 90% accuracy. Thus, by the 12th day of the month, this clinic user would have mastered the therapy tasks and is ready to move on to the next therapy task. In comparison, a home user also starts on the first of the month, takes 6 days to progress from 60 to 90%. This person, however, practices the therapy task every 2 days and needs three sessions to master the task. Thus, by the 6th day of the month, the user would have mastered the therapy task and is ready to move on to the next therapy task. Note that both users complete a median of 20 items to reach 90% accuracy. Thus, these results indicate that the home user practices fewer items per therapy day but practices therapy more often whereas the clinic user practices more items per therapy day but has access to the therapy less often. Overall, these results suggest that similar outcomes can be achieved in home therapy and under the guidance of a clinician, albeit following a different timeframe. There are some advantages to clinic-based treatment, interacting with a clinician hastens the improvement, but conversely patients can progress through their treatment faster when they practice therapy at home more frequently.

There are some important caveats to this retrospective analysis. First, users were not randomly assigned into their groups, they were self-selected in terms of whether they used the app either at the clinic or at home. Therefore, it is possible that severity of the patients may have influenced the results such that more severe users only worked with clinicians whereas less severe patients were able to work independently at home. Our own previous work has shown that severe patients interact with the Constant Therapy platform differently than less severe patients (33). To avoid this problem, we only examined progress from 60 to 90% performance for all patients and Table 4 shows mean starting accuracy levels for each of the tasks analyzed and differences between the two groups. While some differences are significant for some tasks, there are no systemic differences between the two groups and there are also between-task differences in starting accuracy. Another important caveat of this data set is that there are no standardized assessments to validate the improvements made in treatment. This is an important trade off in this type of big data analysis, while large amounts of data sets are collected across several English-speaking countries, it is impossible to collect detailed demographic and assessment information from these individuals. Future work in this area should combine remote assessments that are designed to capture changes made in the treatment program but this data is unavailable for this manuscript.

**Table 4:** Group comparisons of starting accuracy for each task.

**Task name and level**	***t***	**M (sd) of clinic PWA**	**M (sd) of home PWA**
Auditory command 1	0.478	0.48 (0.13)	0.47 (0.13)
Auditory command 1^*^	0.061	0.49 (0.13)	0.48 (0.11)
Auditory command 2	1.576	0.48 (0.11)	0.46 (0.13)
Auditory command 2^*^	−0.047	0.47 (0.14)	0.47 (0.13)
Auditory command 3	−0.32	0.48 (0.12)	0.49 (0.13)
Calendar 1	−2.245^*^	0.42 (0.2)	0.5 (0.15)
Category identification 1	0.617	0.41 (0.16)	0.4 (0.15)
Category matching 1	−2.361^*^	0.49 (0.15)	0.53 (0.11)
Clock math 1	−2.172^*^	0.42 (0.2)	0.48 (0.16)
Clock math 2	−1.597	0.46 (0.17)	0.49 (0.15)
Clock math 3	−1.417	0.42 (0.18)	0.48 (0.16)
Clock reading 1	−0.985	0.45 (0.18)	0.48 (0.16)
Clock reading 2	0.845	0.48 (0.19)	0.44 (0.18)
Currency 1	−1.265	0.41 (0.21)	0.48 (0.15)
Feature matching 1	−0.096	0.49 (0.15)	0.49 (0.14)
Flanker 1	0.6	0.49 (0.15)	0.47 (0.16)
Functional math 1	−2.802^**^	0.34 (0.22)	0.48 (0.14)
Functional reading 1	−2.767^**^	0.45 (0.21)	0.53 (0.12)
Functional reading 2	−2.128^*^	0.49 (0.14)	0.56 (0.11)
Instruction sequencing 1	−0.696	0.46 (0.13)	0.47 (0.12)
Letter to sound matching 1	−1.637	0.43 (0.2)	0.5 (0.14)
Map reading 1	−1.55	0.43 (0.18)	0.48 (0.16)
Mental rotation 1	−0.794	0.5 (0.1)	0.52 (0.12)
Minimal pairs - same or different 1	−0.746	0.45 (0.19)	0.49 (0.13)
Pattern recreation 1	−0.535	0.44 (0.13)	0.45 (0.14)
Pattern recreation 2	−0.902	0.49 (0.08)	0.51 (0.08)
Pattern recreation 3	−0.136	0.48 (0.12)	0.48 (0.11)
Picture matching 2	0.312	0.5 (0.1)	0.5 (0.11)
Picture naming 1	−1.411	0.4 (0.2)	0.46 (0.2)
Picture naming 1^*^	−1.028	0.34 (0.21)	0.38 (0.23)
Picture naming 3	0.803	0.45 (0.22)	0.41 (0.21)
Picture N-back memory 1	−0.654	0.4 (0.17)	0.41 (0.17)
Picture N-back memory 2	−1.015	0.43 (0.16)	0.46 (0.12)
Playing card slapjack 1	−0.001	0.41 (0.18)	0.41 (0.17)
Reading passage 1	−1.311	0.42 (0.21)	0.48 (0.18)
Short reading 1	−3.064^**^	0.43 (0.18)	0.53 (0.12)
Sound identification 1	1.728	0.41 (0.11)	0.37 (0.16)
Sound to letter matching 1	−1.525	0.49 (0.11)	0.53 (0.1)
Spoken word comprehension 1	1.797	0.51 (0.09)	0.46 (0.13)
Symbol matching 6	2.061^*^	0.51 (0.14)	0.44 (0.16)
Voice mail 1	−3.365^**^	0.46 (0.18)	0.52 (0.12)
Voice mail 2	0.453	0.51 (0.15)	0.5 (0.13)
Word identification 1	0.112	0.43 (0.21)	0.43 (0.2)
Word problem 1	−3.076^**^	0.36 (0.22)	0.52 (0.15)
Word repetition 1	−2.001^*^	0.25 (0.24)	0.35 (0.2)
Written word comprehension 1	−0.357	0.48 (0.13)	0.49 (0.09)

In Task Name and Level, *denotes newer versions of a task that were updated and therefore treated as a separate task.

* *p < 0.05*;

** *p < 0.01*.

In consideration of these caveats, this retrospective analysis provides a narrow interpretation of a large data set that directly compares the rate of treatment gains for patients who practice one-on-one in the clinic vs. those who receive therapy only at home. These results contribute to an increasing body of research showing that self-administered computer based therapy and/or telerehabilitation may be a feasible alternate approach to continuing rehabilitation (5, 16, 17, 29–31). Further, the observation that increased therapy practice is associated with more improvement aligns with studies that emphasize the importance of dosage of therapy in chronic aphasia (34–37). For many individuals, visits to a clinician are infrequent and inconsistent which limits the amount of therapy, and thus the recovery they can achieve (10). Remote/computer-based therapy can be practiced daily at home thus allowing patients to practice more intense therapy than periodic visits to a clinic, while achieving similar therapy gains as one-on-one clinician visits. Ultimately, this may facilitate the way the clinicians deliver therapy, which may become more efficient and fulfilling for both the patient and the clinician (38, 39).

## Ethics Statement

This study was carried out in accordance with the recommendations of Pearl IRB. This study was exempt from written informed consent as it was an anonymized retrospective analysis. Our protocol was exempt because (1) the research involves the study of existing data that is recorded by the investigator [per 45-CFR 46.101 (b) (4)], and (2) the research involves the use of education tests (cognitive, diagnostic, achievement), survey procedures that are analyzed in an aggregated form [per 45 CFR 46.101 (b)(2)].

## Author Contributions

VA, MA, SK, and JG conceptualized the project. CD, JG, and SK were involved in analyzing the results and writing the manuscript.

### Conflict of Interest Statement

SK and CD are currently consultants for The Learning Corporation. JG, MA, and VA are employees of The Learning Corporation.
